# Hyperosmolar dehydration: A predictor of kidney injury and outcome in hospitalised older adults

**DOI:** 10.1016/j.clnu.2019.11.030

**Published:** 2020-08

**Authors:** Ahmed M. El-Sharkawy, Mark A.J. Devonald, David J. Humes, Opinder Sahota, Dileep N. Lobo

**Affiliations:** a Gastrointestinal Surgery, Nottingham Digestive Diseases Centre, National Institute for Health Research (NIHR) Nottingham Biomedical Research Centre, Nottingham University Hospitals NHS Trust and University of Nottingham, Queen's Medical Centre, Nottingham, NG7 2UH, UK; b Renal and Transplant Unit, Nottingham University Hospitals NHS Trust, City Campus, Nottingham, NG5 1PB, UK; c Division of Epidemiology and Public Health, University of Nottingham, City Campus, Nottingham NG5 1PB, UK; d Department of Elderly Medicine, Nottingham University Hospitals NHS Trust, Queen's Medical Centre, Nottingham, NG7 2UH, UK; e MRC Versus Arthritis Centre for Musculoskeletal Ageing Research, School of Life Sciences, University of Nottingham, Queen's Medical Centre, Nottingham, NG7 2UH, UK

**Keywords:** Dehydration, Hypohydration, Older adults, Osmolarity, Serum biochemistry, Acute kidney injury

## Abstract

**Background & aims:**

Hospitalised older adults are vulnerable to dehydration. However, the prevalence of hyperosmolar dehydration (HD) and its impact on outcome is unknown. Serum osmolality is not measured routinely but osmolarity, a validated alternative, can be calculated using routinely measured serum biochemistry. This study aimed to use calculated osmolarity to measure the prevalence of HD (serum osmolarity >300 mOsm/l) and assess its impact on acute kidney injury (AKI) and outcome in hospitalised older adults.

**Methods:**

This retrospective cohort study used data from a UK teaching hospital retrieved from the electronic database relating to all medical emergency admissions of patients aged ≥ 65 years admitted between 1st May 2011 and 31st October 2013. Using these data, Charlson comorbidity index (CCI), National Early Warning Score (NEWS), length of hospital stay (LOS) and mortality were determined. Osmolarity was calculated using the equation of Krahn and Khajuria.

**Results:**

A total of 6632 patients were identified; 27% had HD, 39% of whom had AKI. HD was associated with a median (Q1, Q3) LOS of 5 (1, 12) days compared with 3 (1, 9) days in the euhydrated group, *P* < 0.001. Adjusted Cox-regression analysis demonstrated that patients with HD were four-times more likely to develop AKI 12–24 h after admission [Hazards Ratio (95% Confidence Interval) 4.5 (3.5–5.6), *P* < 0.001], and had 60% greater 30-day mortality [1.6 (1.4–1.9), *P* < 0.001], compared with those who were euhydrated.

**Conclusion:**

HD is common in hospitalised older adults and is associated with increased LOS, risk of AKI and mortality. Further work is required to assess the validity of osmolality or osmolarity as an early predictor of AKI and the impact of HD on outcome prospectively.

## 1 Introduction

Increasing age is associated with diminished physiological reserve, resulting in physical as well as functional decline. Age-related pathophysiological changes make the older adult increasingly vulnerable to disturbances in fluid and electrolyte balance, particularly during periods of ill health or physiological stress [1].

Hyperosmolar dehydration (HD), defined as serum osmolality >300 mOsm/kg or osmolarity >300 mOsm/l may be more sensitive for the early detection of dehydration in older adults compared with clinically reported dehydration (based on clinical signs and symptoms of dehydration) due to the challenges in detecting these signs and symptoms in older adults [[2], [3], [4], [5], [6], [7], [8]].

A pilot study of 200 hospitalised older adults assessed the prevalence of HD in prospectively and demonstrated that HD was present in 37% of older adults at admission and was independently associated with mortality [2]. Another study [3] reviewed the electronic records of 32,980 hospital admissions of older adults and demonstrated that clinically reported dehydration (dehydration diagnosed based on clinical assessment) was the primary cause of admission in only 0.6% of patients as a secondary diagnosis 8.9%. Dehydration was associated with a 2.2-fold increase in mortality [3]. Both studies [2,3] reported a higher prevalence of acute kidney injury (AKI) associated with dehydration, but did not clarify whether AKI was a cause or result of dehydration. It is also unclear which of these findings represent a true reflection of the prevalence of dehydration amongst hospitalised older adults and how this affects outcome.

Biochemical markers are thought to be reliable at assessing and monitoring hydration status in this group of patients, with many scientists and clinicians supporting serum osmolality as one of the most accurate objective measures of HD, which is the most common type of dehydration in older adults [[4], [5], [6], [7], [8]]. Despite this, serum osmolality is not measured routinely in clinical practise. However, equations developed to calculate osmolarity have been shown to be accurate enough to be used as a surrogate marker of serum osmolality and are used in the research setting [[9], [10], [11], [12], [13]].

Serum osmolality (mOsm/kg) and calculated osmolarity (mOsm/l) are measures of osmotically active solute concentrations. For a given solution such as plasma the measured osmolality is slightly less than calculated osmolarity, as the denominator used for osmolality includes total mass of solvent and solute, whereas in calculating osmolarity, the solute does not contribute to volume. At physiological plasma solute concentrations, the mass of the solute is small relative to the mass of the solvent, so the difference between measured serum osmolality and calculated osmolarity is negligible and in most cases clinically irrelevant. The most accurate equations calculating osmolarity are based on biochemical markers measured routinely in the clinical setting, including sodium, potassium, urea and glucose [9,10].

Siervo et al. [10] demonstrated that osmolarity calculated using the equation described by Krahn and Khajuria [14] correlated well with measured serum osmolality and had a sensitivity of 97% and a specificity of 79% for predicting HD in community-dwelling older adults.

This study aimed to use the admission records from a large UK university teaching hospital to estimate the prevalence of HD using the equation of Krahn and Khajuria [14] and to assess the impact of HD on AKI, length of hospital stay (LOS) and mortality in hospitalised older adults.

## 2 Methods

**Fig. 1:**
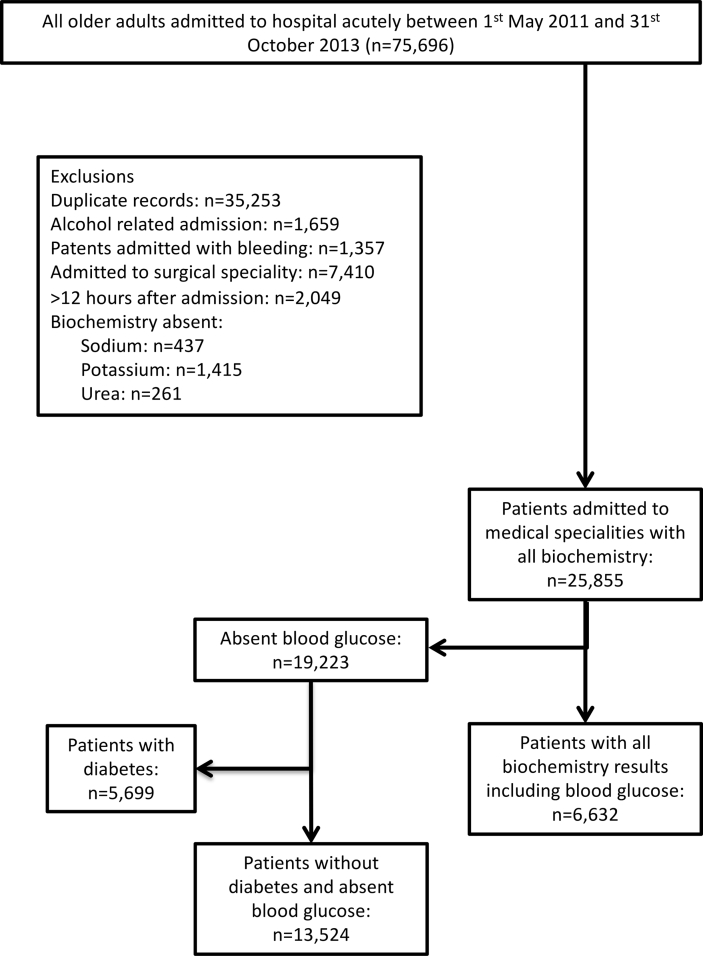
Data selection methods. Patients admitted with any alcohol related condition including alcohol intoxication were excluded to reduce the risk of artificially high osmolar gap, the difference between measured serum osmolality and calculated osmolarity. Patients admitted with bleeding or those admitted to surgery were also excluded. Patients who did not have measured serum biochemistry required for the equation of Krahn & Khajuria within 12 h of admission were also excluded. First admission episode selected. If a patient was admitted multiple times over the study period. Formal laboratory serum glucose measurements were performed on blood sampled used for other biochemistry analysis.

This retrospective cohort study on patients aged ≥65 years was performed using the electronic database of a large UK university teaching hospital. Specialist data analysts searched the hospital's database and retrieved data that related to individual patients aged ≥65 years who were admitted to medical specialities as an emergency between 1st May 2011 and 31st October 2013. Details of the data collected are summarised in Table 1. Each record represented an individual patient with first admission preferentially selected if a patient was admitted multiple times over the study period. Fig. 1 details the cohort selection methods and lists inclusion/exclusion criteria.

**Table 1:** Details of the data collected from the hospitals database and the confounders used in the analysis.

Data retrieved	Details	Notes
Unique identification (ID) code and demographics	Hospital unique ID, date of birth, age & gender.	Gender^c^ Age was categorised into four time periods: 65 to 75, 76 to 85, 86 to 95 and > 95 years^c^
International Classification of Disease, 10th Revision, 4th edition ICD-10 diagnoses codes	Up to 25 diagnoses related to the admission and comorbidity classified according to the ICD-10	Charlson Comorbidity Index^c^ Score (CCI) was calculated using all diagnoses codes retrieved utilising the scoring protocol developed by Charlson et al. (J Clin Epidemiol 1994; 47:1245-51) to account for comorbidities
Serum biochemistry & osmolarity^a^	Serum sodium, potassium, urea, creatinine, glucose & osmolality	Osmolarity was calculated using the equation of Krahn & Khajuria [1.86 × (sodium^b^ + potassium)^b^ + (1.15 × glucose)^b^ + urea^b^ + 14]
Date and time of acute kidney injury (AKI) diagnosis^b^	Risk of AKI based on change in creatinine >26.5 μmol/l	The risk of AKI 12–24 h, 12–48 h and 12–72 h post admission was calculated and patients with AKI at admission to hospital (12 from time of admission) were excluded for this analysis
Dates of admission & discharge	Used to calculate length of hospital stay (LOS)	
Dates of death	Used to calculate in-hospital, 30, 90 and 365 days (one-year) post-admission mortality	Data available regardless of whether patients had died in hospital or in the community, up to 29th December 2014
Observations	Pulse rate, blood pressure, temperature, oxygen saturations, level of consciousness	National early warning score^c^ (NEWS) was calculated in accordance with published guidelines to account for the severity of illness at admission for a subset of patients who had electronically reported clinical observations at admission to hospital

a Analysed at the hospital's clinical pathology laboratory.

b Data were linked using the patients' unique hospital number and date of admission. Full details of the algorithm used for the alert have been published by Porter et al. [16].

c Confounders used in the analysis.

The hospital's databases are updated and crosschecked with the National Summary Care Record system by the Data Quality team daily. Regular internal audits are undertaken by a Health and Social Care Information Centre which reported that primary and secondary diagnoses were 96% and 93%–95% accurate, respectively. The dataset used in the present study was also used to investigate the prevalence of clinically diagnosed dehydration and its impact on outcome [3]. However, the present study used biochemical indicators of dehydration to investigate HD in older adults.

HD was defined as osmolarity >300 mOsm/l calculated using the equation described by Krahn and Khajuria [14] for patients with relevant serum biochemistry results. Units for all values used in the equation were mmol/l.

Serumosmolarity(mOsm/l)=[1.86×(sodium+potassium)+(1.15×glucose)+urea+14]

Clinically reported dehydration (ICD-10 code E86.X) indicated severe dehydration or dehydration requiring intravenous fluids. AKI was defined using a combination of criteria from Risk, Injury, Failure, Loss of kidney function, and End-stage kidney disease (RIFLE) and Acute Kidney Injury Network (AKIN) classifications, widely accepted as the diagnostic benchmark at the time of the data collection [15]. In effect, the criteria were very similar to the subsequently internationally-adopted KDIGO classification system and the algorithms used have been published RIFLE classifications “risk ”and “injury” were considered equivalent to AKI stage 1 and 2 respectively [16]. Whilst “failure”, “loss of function” and “end stage kidney disease” were classified as stage 3 [16]. Diagnosis and stage of AKI were retrieved from the hospitals AKI electronic-alert database and linked using the patients’ unique hospital number and date of admission [16].

### 2.1 Statistical analysis

Data analysis was performed using Stata Statistical Software, Release 13 (StataCorp., College Station, TX, USA). Distribution plots were used to assess the distribution of continuous data. Data that were distributed normally were presented as means and standard deviations (SD) and independent samples *t*-test was used to assess for significant differences. Non-parametric data were presented as medians and interquartile ranges (Q1, Q3) and the Kruskal-Wallis test was used to assess for differences.

Cox regression modelling was used to provide unadjusted and adjusted hazard ratios (HR) as an approximation of risk of AKI as well as mortality in the presence of dehydration. Multivariate analysis was used to adjust for potential confounding factors such as age, gender and comorbidities (Charlson Comorbidity Index – CCI). Further adjustment for National Early Warning Scores (NEWS) was performed for a subset of patient records that contained the relevant information to allow this score to be calculated in order to account for severity of illness at admission (Table 1). Kaplan-Meier plots were generated to schematically represent mortality over time.

## 3 Results

### 3.1 Prevalence of dehydration

**Fig. 2:**
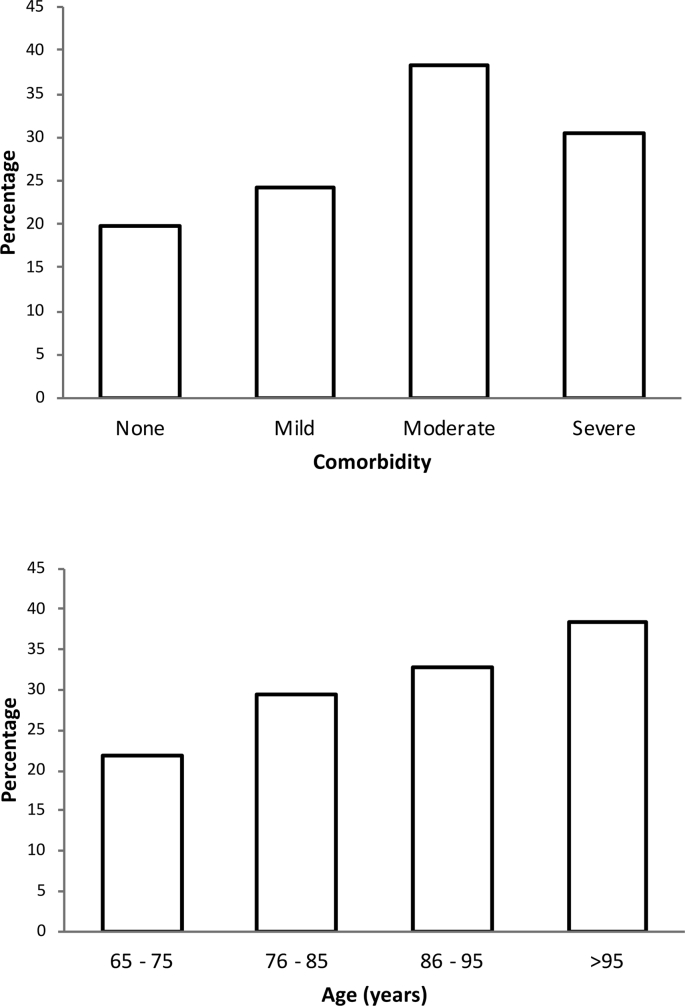
(top) Prevalence of dehydration with Charlson Comorbidity Index (age unadjusted). ‘None’ (no comorbidity, 0 points), ‘Mild’ (mild comorbidity, 1–2 points), ‘Moderate’ (moderate comorbidity, 3–4 points) and ‘Severe’ (severe comorbidity, ≥5 points). (bottom): Prevalence of dehydration with increasing age.

A total of 6632 patients had all the serum parameters required for the equation of Krahn and Khajuria [14] to be calculated at admission to hospital (Fig. 1). Patient characteristics are summarised in Table 2. HD was noted in 1802 (27.2%) patients at admission. Clinically diagnosed dehydration, however, was reported in only 676 (10.2%) patients. Of the 1802 patients with HD, 313 (17.4%) were diagnosed with dehydration by the clinical team compared with 363 (7.5%) patients in the biochemically euhydrated group (n = 4830), *P* < 0.001. The mean (SD) osmolarity in patients with HD who were clinically dehydrated was 319 (22) compared with 308 (12) in those with HD who were not clinically dehydrated, *P* < 0.001. The mean (SD) age of patients with HD was 79.0 (8.3) years compared with 77.6 (8.1) years in euhydrated patients. The median CCI (Q1, Q3) scores in patients with and without HD were 1 (1, 2) and 1 (0, 2) respectively, *P* < 0.001. Stratification by age and CCI revealed that the prevalence of dehydration increased with age and comorbidities (Fig. 2).

**Table 2:** Demographics and characteristics of the study cohort. Comparing those with and without hyperosmolar dehydration.

		All Patients (n = 6632)	Euhydrated (n = 4830)	Dehydrated^b^ (n = 1802)	*P* value^a^
Age (years)	65–75: n (%)	2692 (40.6)	2103 (43.5)	589 (32.7)	<0.001
	76–85: n (%)	2555 (38.5)	1801 (37.3)	754 (41.8)	
	86–95: n (%)	1286 (19.4)	865 (17.9)	421 (23.4)	
	>95: n (%)	99 (1.5)	61 (1.3)	38 (2.1)	
Gender	Female: n (%)	3469 (52.3)	2596 (53.7)	873 (48.4)	<0.001
	Male: n (%)	3163 (47.7)	2234 (46.3)	929 (51.6)	
Charlson Comorbidity index	None (0): n (%)	1135 (17.1)	910 (18.8)	225 (12.5)	<0.001
	Mild (1-2): n (%)	3117 (47.0)	2364 (48.9)	753 (41.8)	
	Moderate (3-4): n (%)	1265 (19.1)	780 (16.1)	485 (26.9)	
	Severe (≥5): n (%)	1115 (16.8)	776 (16.1)	339 (18.8)	
Admission Method	Emergency Department: n (%)	2496 (37.6)	1902 (39.4)	594 (33.0)	<0.001
	General Practitioner: n (%)	3626 (54.7)	2522 (52.2)	1104 (61.3)	
	Other: n (%)	510 (7.7)	406 (8.4)	104 (5.8)	

Osmolarity calculated using the equation of Krahn & Khajuria [1.86 × (sodium + potassium) + (1.15 × glucose) + urea +14].

a *P* value comparing patients with and without dehydration.

b Dehydration indicates hyperosmolar dehydration, serum osmolarity >300 mOsm/l.

### 3.2 Dehydration and AKI

AKI was diagnosed in 710 (39.4%) patients with HD, compared with 818 (16.9%) patients who were biochemically euhydrated, *P* < 0.001. Moreover, a greater proportion of those with HD had more severe AKI, compared with those without HD, AKI stage 2: 160 (8.9%) *vs.* 145 (3.0%) and AKI stage 3: 162 (9.0%) *vs.* 63 (1.3%), respectively, *P* < 0.001. Patients with HD at admission to hospital were at increased risk of developing AKI within 24 h, independent of age, gender and CCI [Hazards Ratio (HR) (95% Confidence Interval - CI) 4.45 (3.53–5.60), *P* < 0.001]. The risk of AKI was also independently greater 48 and 72 h after admission in patients with HD compared with the euhydrated group (Table 3a). Further analysis demonstrated that the risk of AKI associated with HD was also independent of NEWS (Table 3b).

**Table 3:** Hyperosmolar dehydration, acute kidney injury and mortality.

	*(a) Whole cohort (n*=*6632)*							(b) Patients with national early warning score (n = 422)						
	Euhydrated (n = 4830)	Dehydrated^a^ (n = 1802)	*P* value	Unadjusted: HR (95% CI)	*P* value	Adjusted^b^: HR (95% CI)	*P* value	Euhydrated (n = 274)	Dehydrated^a^ (n = 148)	*P* value	Unadjusted: HR (95% CI)	*P* value	Adjusted^c^: HR (95% CI)	*P* value
All AKI	818 (16.9)	710 (39.4)	<0.001	–	–	–	–	54 (19.7)	58 (39.2)	<0.001	–	–	–	–
12–24 h post admission	119 (2.5)	203 (11.3)	<0.001	4.79 (3.82 to 6.01)	<0.001	4.45 (3.53 to 5.60)	<0.001	6 (2.2)	16 (10.8)	<0.001	5.13 (2.01 to 13.12)	0.001	5.15 (1.8 to 14.64)	0.002
12–48 h post admission	212 (4.4)	266 (14.8)	<0.001	3.59 (3.00–4.30)	<0.001	3.28 (2.73–3.94)	<0.001	15 (5.5)	22 (14.9)	0.001	2.90 (1.50–5.58)	0.001	2.74 (1.32–5.70)	0.007
12–72 h post admission	272 (5.6)	303 (16.8)	<0.001	3.22 (2.73–3.79)	<0.001	2.93 (2.48–3.46)	<0.001	22 (8.0)	29 (19.6)	0.001	2.64 (1.52–4.60)	0.001	2.54 (1.38–4.64)	0.003
In-hospital mortality	381 (7.9)	231 (12.8)	<0.001	2.09 (1.75–2.48)	<0.001	1.72 (1.44–2.06)	<0.001	16 (5.8)	20 (13.5)	0.007	2.81 (1.23–6.42)	0.014	1.82 (0.94–3.53)	0.077
30-day mortality	381 (7.9)	265 (14.7)	<0.001	1.93 (1.65–2.26)	<0.001	1.61 (1.36–1.89)	<0.001	18 (6.6)	22 (14.9)	0.005	3.40 (1.62–7.14)	0.001	1.92 (1.03–3.56)	0.039
90-day mortality	679 (14.0)	306 (17.0)	<0.001	1.69 (1.50–1.92)	<0.001	1.40 (1.24–1.60)	<0.001	26 (9.5)	27 (18.2)	0.009	4.42 (2.40–8.15)	<0.001	1.63 (0.96–2.78)	0.071
One-year mortality	1224 (25.3)	629 (34.9)	<0.001	1.49 (1.36–1.65)	<0.001	1.23 (1.11–1.36)	<0.001	56 (20.4)	44 (29.7)	0.028	3.19 (1.91–5.33)	<0.001	1.28 (0.85–1.91)	0.234

a Dehydration indicated hyperosmolar dehydration, osmolarity >300 mOsm/l.

b Adjusted for age, gender, Charlson comorbidity index.

c Adjusted for age, gender, Charlson comorbidity index and National Early Warning Score (NEWS).

### 3.3 Dehydration and mortality

**Fig. 3:**
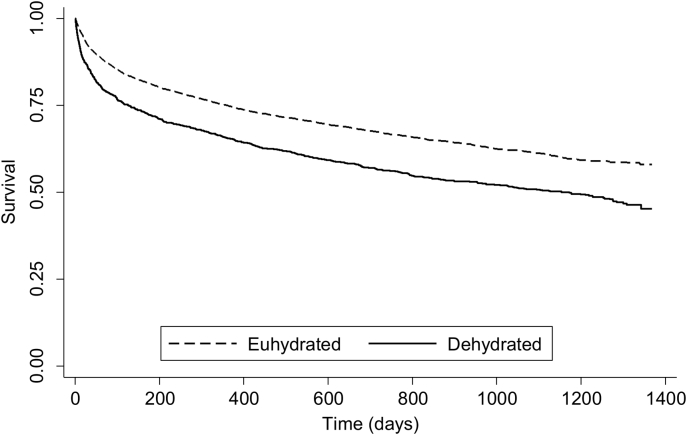
Kaplan-Meier survival plot demonstrating the relationship between hydration status and mortality (*P* < 0.001). Dehydrated indicates hyperosmolar dehydration.

Mortality rates at all time periods were consistently higher in patents with HD (Table 3). The Kaplan-Meier survival plot demonstrates a significant drop in survival after admission in those diagnosed with HD (Fig. 3). Cox regression analysis adjusted for age, gender and comorbidity (CCI) demonstrated that patients with HD were at higher risk of 30-day mortality compared with those who were euhydrated at admission [HR (95% CI) 1.61 (1.36–1.89), *P* < 0.001] (Table 3a). Further analysis revealed that dehydration-related 30-day mortality was also independent of NEWS (Table 3b).

### 3.4 Dehydration and length of hospital stay

The median LOS excluding in-hospital mortality was 5 (1, 12) in patients with HD compared with 3 (1, 9) in the euhydrated group, *P* < 0.001.

## 4 Discussion

### 4.1 Prevalence of dehydration

This study has shown that HD occurred in over a quarter of hospitalised older adults, consistent with previously reported results using osmolality as the marker of hydration [2]. However, the prevalence of HD in the present study was significantly greater than previous reports that utilise clinically reported dehydration [2,3,17]. These significant differences between clinically reported dehydration and HD are most likely to be the result of limited sensitivity of the clinical features of dehydration in this age group as well as the lack of screening and poor documentation [1,[4], [5], [6], [7], [8]].

The present study also demonstrated increased prevalence of dehydration with increased age and comorbidity consistent with previous reports [2,3,17], and is a likely consequence of age-related pathophysiological changes, increased comorbidity and polypharmacy, all of which result in physiological vulnerability and homeostatic irregularity as well as organ dysfunction [1]. However, given the high prevalence of HD at admission, chronic or pre-existing dehydration should also be considered as a contributing factor. Studies have demonstrated that up to 60% of seemingly “well” community-dwelling older adults may be dehydrated [12,13].

In the present study, 39% of those who had HD also developed AKI with a significantly greater proportion that had advanced (stage 3) AKI compared with those who were euhydrated. Moreover, regression analysis revealed patients with HD were over five times more likely to have AKI 12–24 h after admission, adjusted HR (95% CI) 5.15 (1.8–14.64), *P* < 0.001, independent of age, gender, comorbidity and illness severity (NEWS). It is, of course, difficult to attribute cause and effect in this retrospective study and as definitions of AKI are based on arbitrary proportional changes in serum creatinine, it is not surprising that HD correlates with incidence and severity of AKI. However, considering the age-related pathophysiological and renal changes [1,[18], [19], [20], [21]], recognising and treating dehydration may help prevent AKI in some patients, which might in turn improve some of the poor outcomes associated with AKI including increased mortality, length of hospital stay and fall in baseline renal function [22,23]. Treatment of dehydration is of particular importance where patients are prescribed diuretics and nephrotoxic drugs, a common occurrence in older adults.

Adverse events related to diuretics account for up to 25% of all adverse drug reactions in older adults, mostly due to poor monitoring and difficulties in accurately assessing hydration status [[24], [25], [26]]. Currently, many clinicians rely on changes in blood urea and serum creatinine concentrations to aid the assessment of hydration status and fluid balance. However, these are not sensitive to small changes in hydration status, and are themselves markers of AKI [25,27]. Moreover, there is evidence suggesting that changes in creatinine concentration may lag several days behind actual changes in glomerular filtration rate [28,29]. Therefore, the results for the present study lend support to the use of serum osmolality to facilitate the diagnosis of HD as well as helping with prescription of diuretics and high-risk drugs. This approach may help prevent AKI, which is also independently associated with high morbidity and mortality [16,22,23].

HD was also found to be associated with mortality. The 30-day mortality in the dehydrated group was nearly twice that of the euhydrated group, 14.7% *vs.*7.9% respectively, *P* < 0.001. It was also associated with a greater risk of 30-day and 90-day mortality, independent of age, gender and comorbidity. These findings are consistent with previous reports [2] and are unlikely to be unique to this cohort or this centre which was described by the Care Quality Commission (CQC) as “safe, caring, effective and well-led” and had a standardised mortality rate in keeping with the national average [30,31]. Studies in other centres have also shown that high osmolality at admission was associated with increased morbidity and mortality after ischaemic stroke and myocardial infarction [[32], [33], [34]]. The higher mortality rates may be related to complications of dehydration such as AKI as well as other organ dysfunction [35]. The complications associated with dehydration may also explain the marked increase in LOS associated with HD reported in this study [35]. This, will further contribute to financial and resource pressures already facing the health service.

### 4.2 Limitations

This study reports significant findings with potential clinical implications. There are some limitations that need to be considered when interpreting the results. HD (serum osmolality >300 mOsm/kg) is thought to be the most common form of dehydration in older adults, equivalent to a reduction of between 4 and 5% of body weight [36,37]. However, this does not represent all forms of dehydration and it is, therefore, important to consider salt and water balance when using serum osmolality to assess hydration status [[4], [5], [6], [7]].

Although osmolarity calculations have been shown to be comparable to measured serum osmolality and were also highly sensitive and specific at predicting HD [10], osmolarity is only an estimation of hydration status and may be influenced by various factors including serum alcohol concentrations. Efforts were made to exclude all alcohol-related admissions to minimise bias (Fig. 1).

HD may be a manifestation of disease severity, and an increase in LOS and mortality would, therefore, be expected. However, we have attempted to account for confounders including age, gender and comorbidities using the CCI and NEWS.

LOS analysis was based on admission and discharge dates and not the date the patient was medically fit for discharge. The LOS results should, therefore, be considered with caution as delayed discharges are common in older adults, a result of complex discharge processes, which may attenuate or exacerbate differences in LOS reported in this study. However, it may be reasonable to assume that the incidence of delayed discharge may be equal between dehydrated and euhydrated patients.

The findings of the present study are based on data obtained from a large university teaching hospital. Further work is required to assess whether these findings are applicable to other hospitals. However, there are numerous reports (although less comprehensive) from hospital and community settings suggesting that fluid mismanagement may be more widespread [38,39].

## Funding

This work was supported by the Medical Research Council, UK [grant number MR/K00414X/1]; and Arthritis Research UK [grant number 19891]. AME-S was funded by a research grant from the European Hydration Institute, Madrid, Spain. The funders had no role in the design of the study, collection or analysis of data, writing of the manuscript or decision to submit for publication.

## Author contributions

Study design: AME-S, MAJD, DJH, OS, DNL.

Data collection and analysis: AME-S, MAJD, DJH.

Data interpretation: AME-S, MAJD, DJH, OS, DNL.

Writing of manuscript: AME-S, MAJD, DJH, OS, DNL.

Critical revision: AME-S, MAJD, DJH, OS, DNL.

Final approval: AME-S, MAJD, DJH, OS, DNL.

## Conflict of interest

None of the authors has a direct conflict of interest to declare. DNL has received unrestricted research funding from B. Braun and speaker's honoraria from Fresenius Kabi, B. Braun, Shire and Baxter Healthcare for unrelated work.
